# Anterior Cruciate Ligament (ACL) Injury Mechanisms in Men’s Major League Soccer: A Systematic Video Analysis

**DOI:** 10.7759/cureus.110002

**Published:** 2026-05-31

**Authors:** Casey A Clarke, Shawn A Moore, Ronak J Mahatme, Anish Gangavaram, Nishanth Muthusamy, Paul P McMillan, Brian M Grawe

**Affiliations:** 1 Department of Orthopaedic Surgery, University of Cincinnati College of Medicine, Cincinnati, USA; 2 Division of Sports Medicine, Department of Orthopaedic Surgery, University of Cincinnati College of Medicine, Cincinnati, USA

**Keywords:** anterior cruciate ligament (acl), injury prevention, major league soccer, professional athletes, sports injuries

## Abstract

Introduction

Anterior cruciate ligament (ACL) injuries are common in professional soccer; however, limited research has described the situational mechanisms of injury in men's major league soccer (MLS) in the United States and Canada. Understanding these mechanisms is essential for developing targeted prevention and rehabilitation strategies.

Objective

This study aims to identify situational patterns and positional characteristics associated with ACL injuries in MLS players.

Methods

Across five and a half MLS seasons, 65 ACL injuries were identified. Video footage was available for 33 injuries, which were analyzed to determine the mechanism of injury, player activity at the time of injury, knee position, and external factors, including field type, month of injury, and timing within the game.

Results

Non-contact injuries were most common (64%), followed by direct (21%) and indirect-contact (15%) injuries. Injuries most frequently occurred during defensive pressing (76%) and offensive driving (58%). Knee valgus (82%) and flexion (85%) were the predominant positions at the time of injury. All indirect-contact injuries involved force applied to the upper body, increasing stress on the knee. Injuries occurred more commonly on grass fields during the second half or overtime (61%) and demonstrated a bimodal distribution across the season. Overall, 79% of ACL injuries occurred without direct contact to the affected knee.

Conclusion

ACL injuries in MLS primarily result from non-contact or indirect-contact mechanisms, often during pressing or driving movements, with the knee loaded in valgus and flexion. These findings can inform sport-specific prevention strategies aimed at reducing ACL injury risk and prolonging athlete participation in professional soccer.

## Introduction

Ligamentous knee injuries are common across competitive and club-level sports, sidelining athletes each season. Among these, anterior cruciate ligament (ACL) ruptures are particularly devastating, especially in professional soccer, where they often lead to prolonged absences and even career-ending consequences. Despite increased research efforts, ACL injuries remain a substantial and growing concern in professional soccer [1,2]. The incidence of ACL injury has been estimated at nearly one injury per 10,000 athletic exposures (AE), defined as one athlete participating in one practice or competition, in male soccer players, with rates approximately twice in female soccer players [1,2].

While global studies have examined ACL injuries in soccer [3,4], limited research has focused on major league soccer (MLS), which includes 30 professional teams in the United States and Canada [5,6]. Moreover, the risk factors contributing to ACL rupture remain incompletely understood. Although most tears occur through non-contact mechanisms, emerging evidence suggests that indirect contact may also play a role [7]. Additional contributors may include prior ACL injury, player workload, and the timing of injury within a season.

The purpose of this study was to identify situational patterns of ACL injury in MLS, focusing on player position, seasonal distribution, and the presence of contact at the time of injury. Understanding such factors is critical for designing prevention and rehabilitation strategies. Standardizing programs that emphasize dynamic stretching and targeted strengthening of the supporting musculature may reduce ACL injury rates, prolong athletic careers, and decrease the long-term risk of osteoarthritis and other sequelae [8,9]. We hypothesized that ACL injuries in MLS players would most commonly occur through non-contact or indirect-contact mechanisms. Additional analyses regarding seasonal clustering, positional characteristics, and situational patterns were considered exploratory and descriptive.

## Materials and methods

Injury search

A systematic search for ACL tears among MLS players was performed across five and a half seasons (January 2020 to July 2025). Injuries were included if they occurred in players from any of the 30 MLS teams, MLS lower-division affiliates, or MLS players on loan to international teams. Both first-team MLS players and MLS-affiliated or loaned players were included if the ACL injury occurred during participation in organized match competition. Injuries sustained outside MLS competition, including international matches and non-MLS-affiliated play, were included when verifiable video footage and injury confirmation were available.

ACL injuries were first identified using Transfermarkt.de [10]. Each team roster from 2020 to 2025 was reviewed, and every player’s injury history was searched for “cruciate knee injury.” If the ACL was not specified, additional searches of local and national news sources were performed to determine the specific ligament involved. Any nonspecific “knee injury” designations were further investigated in the same manner. ACL injury diagnoses identified through Transfermarkt were cross-referenced with official team announcements, league reports, and local or national media coverage whenever available to improve diagnostic accuracy and reduce misclassification. This methodology follows prior studies of ACL injuries in professional football and Italian league soccer [11].

To ensure completeness, supplemental searches included MLS team websites and local news reports. In total, 65 ACL tears were identified between January 2020 and July 2025. Institutional Review Board approval was not required for this study, and no consent was required for this systematic video review.

For injuries occurring in 2023 or later, a video review was performed using Apple TV (Apple Inc., Cupertino, CA) [12]. If footage was unavailable, other sources (e.g., ESPN, YouTube, and MLSsoccer.com [13-15]) were used. Injuries sustained during training were excluded, as they were not systematically recorded by teams. Video footage from preseason and international tournament matches could not be identified. High-quality video was available for 33 of the 65 ACL tears. Each injury clip was cut to ~10 seconds before and 5 seconds after the event and reviewed using desktop software.

Video review

Each video was reviewed independently by two reviewers, including an orthopedic surgery resident, to document the situational aspects of injury. Videos were analyzed using QuickTime Player (Apple Inc., Cupertino, CA) frame-by-frame review after extraction from the original broadcast footage. Disagreements regarding mechanism classification, knee position, or player activity were resolved by consensus review. Reviewers were not blinded to player or injury information. Formal inter-rater reliability analysis was not performed, given the descriptive nature of the study.

The mechanism of injury was classified as non-contact, indirect contact, or direct contact. Non-contact injuries were defined as those occurring without prior player-to-player interaction. Indirect-contact injuries were those in which contact occurred at a body site other than the injured knee, with the location of the contact specified. Direct-contact injuries were defined by an external force applied directly to the affected knee.

Finally, the position of the knee at the time of injury was determined and categorized as either flexion or extension and valgus or varus alignment. A “loaded” knee was defined as a position in which the majority of the player’s body weight appeared to be supported by the injured extremity at the time of injury. Videos were slowed as needed to ensure accurate evaluation of player actions, injury mechanism, and knee position. Still frames were extracted from match footage for illustrative purposes; image resolution was limited by the quality of the original broadcast video.

## Results

Match analysis

Out of 65 recorded ACL injuries, 33 had available footage for analysis. All occurred in dry field conditions. Fourteen injuries (42%) took place in the first half of the season (January-June) and 19 (58%) in the second half (July-December). Most injuries occurred on natural grass fields (23/33, 70%), followed by turf (9/33, 27%) and hybrid fields (1/33, 3%), which are 95% grass and 5% synthetic. Out of the 30 total MLS fields, 21 are natural grass fields, six are a variation of turf, and three are hybrid fields. Regarding timing within matches, 11/31 (35%) injuries occurred in the first half, 16/31 (52%) in the second half, and 4/31 (13%) in overtime (timing unavailable for two cases).

Of the 33 players with analyzed injuries, eight (24%) players had a history of prior ACL tear (one with two previous tears). One player (3%) suffered a retearing of the same ACL, while six players (18%) sustained contralateral tears, and the laterality was unclear for an additional player. One player tore the contralateral ACL in subsequent seasons, while one suffered a retearing of the same ACL. All injuries required surgery.

Injury-specific analysis

**Figure 1:**
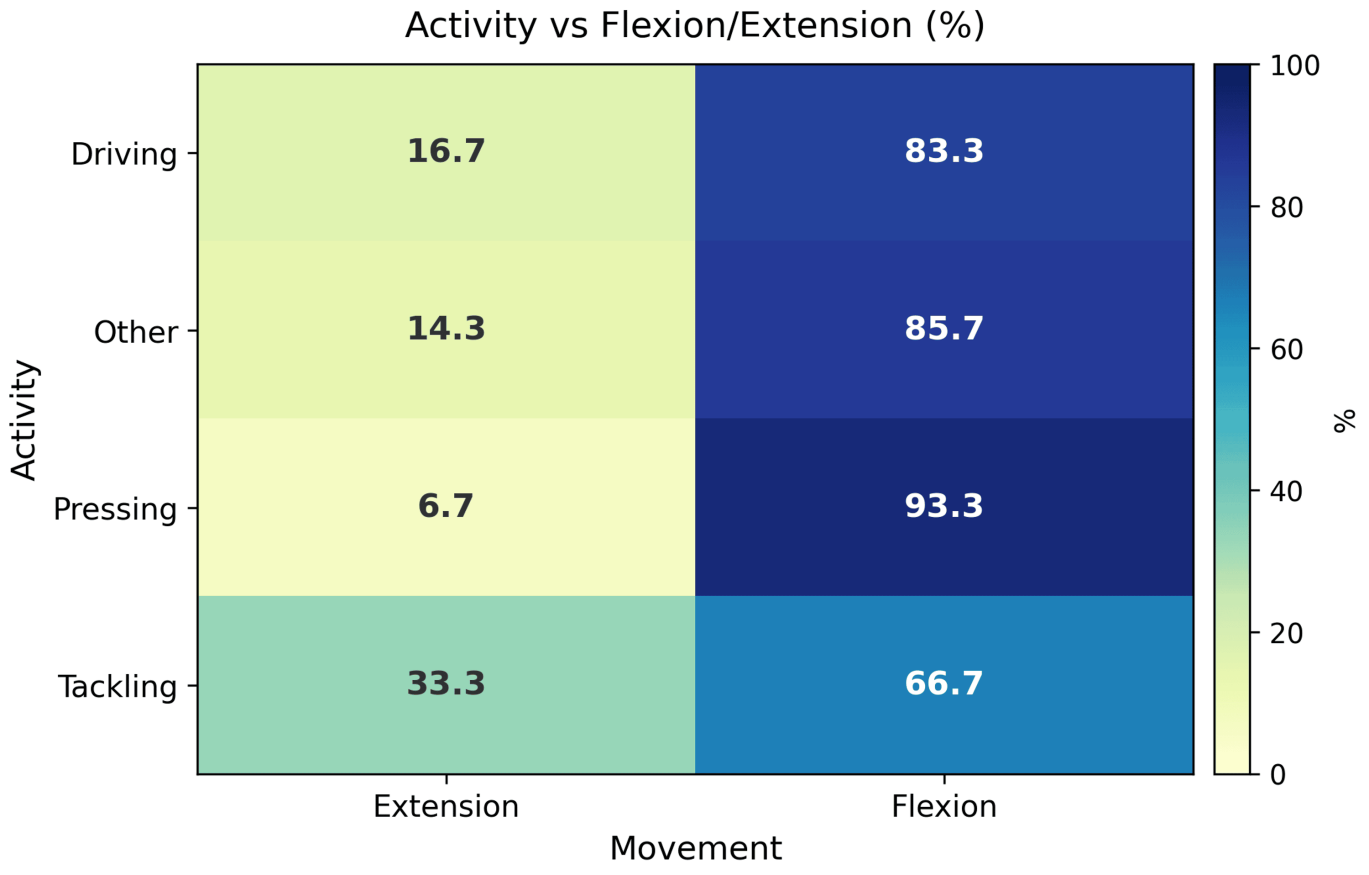
Distribution of knee flexion and extension during ACL injury events by player activity Heatmap demonstrating the percentage of ACL injuries occurring with the knee in flexion or extension during different soccer-related activities in major league soccer players. ACL: Anterior cruciate ligament.

**Figure 2:**
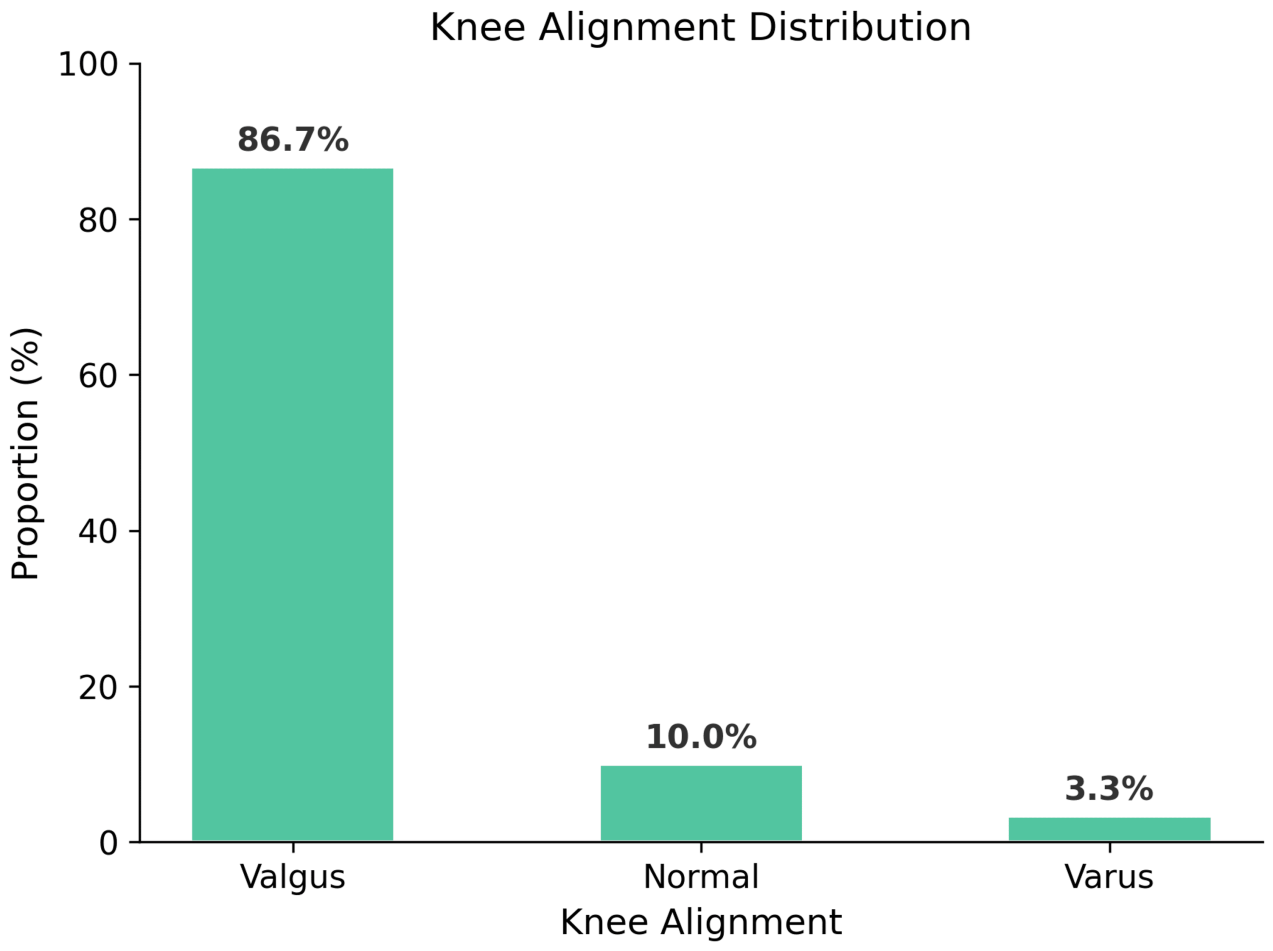
Knee alignment at the time of ACL injury Of the 33 ACL injuries evaluated in this study, 27 (82%) occurred with the knee in valgus alignment, most commonly during offensive cutting or defensive pressing maneuvers. ACL: Anterior cruciate ligament.

Injuries were nearly evenly distributed between knees (15 right and 18 left). At the injury frame, 27/31 (88%) ACLs were loaded (two uncertain), and 28/33 (85%) involved a single-leg stance. Two cases (6%) occurred with both feet on the ground, while two were indeterminate. Overall, 28 (85%) injured knees were in flexion, and 27 (82%) were in valgus alignment (Figures 1, 2).

When stratified by role, 12 (36%) injuries occurred on offense and 21 (64%) on defense. On offense, seven (58%) occurred while driving the ball, with the remainder during shooting, jumping, or being tackled. On defense, 16 (76%) occurred during pressing, with the remainder occurring during tackling or jumping. Preceding contact to the upper body was present in nine (27%) cases, most often involving the arms during ball challenges.

Direct-contact injuries

Direct-contact injuries accounted for seven (21%) cases, including 5/7 (71%) on defense and 2/7 (29%) on offense. Of these injuries, 4/7 (57%) occurred during tackling/being tackled, 2/7 (29%) during pressing collisions, and 1/7 (14%) while an offensive player was driving the ball. Overall, 6/7 (86%) occurred with the knee in valgus; 3/7 (43%) were in extension, and 4/7 (57%) were in flexion.

Indirect-contact injuries

**Figure 3:**
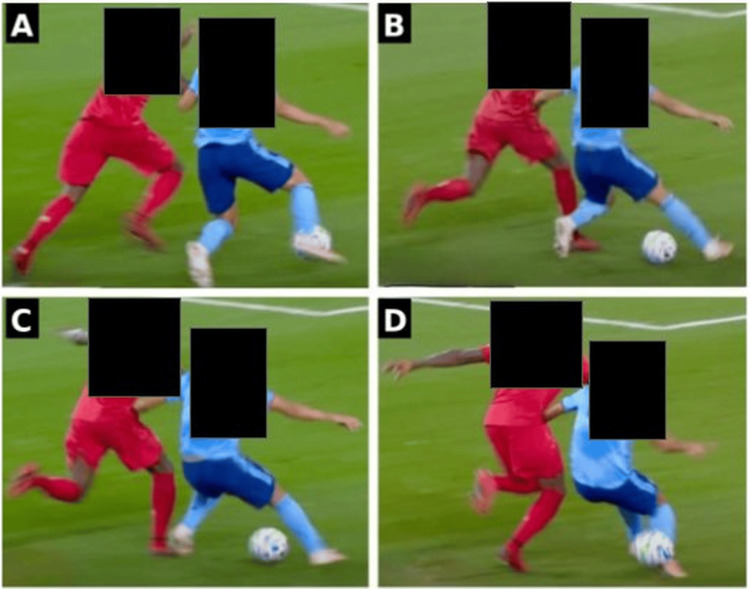
Indirect-contact ACL injury during defensive pressing Sequential video frames demonstrating an indirect-contact ACL injury occurring during a defensive pressing maneuver in a major league soccer player. (A) The defender initiates upper-body contact, while the offensive player begins a cutting movement. (B) Continued upper-body contact shifts the offensive player’s balance during directional change. (C) The planted right knee enters flexion and dynamic valgus while rotational force is applied. (D) Peak valgus collapse and flexion of the right knee occur immediately before ACL injury. Note: Image quality reflects the sequential video frames from which these stills were captured; blurriness is inherent to the source footage and not a result of image processing. ACL: Anterior cruciate ligament.

Indirect-contact injuries represented five (15%) cases. In 4/5 (80%) of these injuries, the indirect force was applied to the upper body, while 1/5 (20%) involved force to the uninjured leg. Activities at the time of injury included pressing (2/5, 40%), driving (2/5, 40%), and tackling (1/5, 20%). Additionally, four (80%) of these injuries occurred with the knee in flexion and valgus alignment (Figure 3).

Non-contact injuries

Non-contact injuries were the most common, comprising 21 (64%) cases. Two injuries were excluded from positional analysis due to poor video quality, leaving 19 evaluable injuries. The most frequent activity was pressing (12/21, 57%), followed by driving (4/21, 19%), shooting (2/21, 9%), jumping (2/21, 9%), and running (1/21, 5%). Flexion was present in nearly all cases, while valgus was observed in 16/19 (84%) injuries, with one varus and two neutral alignments.

Seasonal distribution

**Figure 4:**
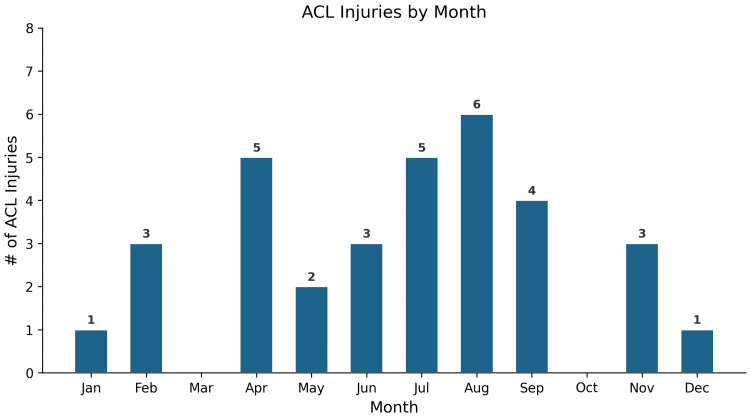
Seasonal distribution of ACL injuries Bar graph demonstrating the monthly distribution of ACL injuries in major league soccer players across the calendar year. ACL injuries clustered at the beginning and end of the MLS season, corresponding to early-season competition and tournament play later in the season. ACL: Anterior cruciate ligament.

ACL injuries demonstrated a bimodal distribution across the MLS calendar year. Injuries were more frequently observed during periods of increased workload and competition intensity (Figure 4).

## Discussion

In this study of ACL injuries in MLS over five and a half seasons, most tears were classified as non-contact (21/33, 64%), with indirect contact accounting for 5/33 (15%) and direct contact accounting for 7/33 (21%) injuries. The majority of injuries occurred when the knee was in flexion (28/33, 85%) and valgus alignment (27/33, 82%), and the most common activities at the time of injury were driving the ball on offense and pressing on defense. Injuries were slightly more frequent in the second half of games or during overtime (20/33, 61%) and demonstrated clustering near the beginning and end of the season.

These findings align with prior studies in soccer and other sports, such as American football, where non-contact or indirect-contact mechanisms predominate [7]. Indirect-contact injuries, often involving force to the upper body during player challenges, can contribute to valgus stress on the knee, further loading the ACL. Rapid cutting and directional changes, such as driving the ball toward the goal or pressing an opponent, appear to place the knee at high risk, as these movements often load the leg in flexion and valgus [16-18]. Understanding these situational mechanisms may help guide future sport-specific ACL prevention research incorporating dynamic soccer-specific movements and directional-change mechanics.

ACL injuries demonstrated clustering near the beginning and end of the MLS season. Although this pattern may relate to changes in training intensity, match congestion, return-to-play demands, or tournament scheduling, the present study did not directly measure workload or athlete exposure. Therefore, these findings should be interpreted as descriptive and hypothesis-generating rather than causal. Nearly one-third of injured players had a prior history of ACL tear, reinforcing previous injury as a known risk factor for subsequent ACL injury. Additionally, a substantial number of identified ACL injuries occurred during preseason or training settings, although these cases were excluded from video analysis because of limited footage availability.

Existing programs such as FIFA’s 11+ and the PEP (Prevent Injury and Enhance Performance) program have demonstrated efficacy in reducing ACL injury risk, particularly when emphasizing eccentric and single-leg movements [19-21]. However, our data may help guide future MLS-specific prevention research focused on cutting, pressing, pivoting, and other high-risk soccer movements observed in professional players. Many injuries occurred at the start of the season, when players may be returning from periods of reduced competition exposure, although workload and conditioning data were not directly measured in this study [22-25].

Game timing was also notable, with 20/33 (61%) ACL injuries occurring in the second half or overtime, raising the possibility that fatigue or cumulative match demands may contribute to injury risk. However, fatigue was not directly measured in this study. Future research should evaluate whether cumulative minutes played, workload metrics, or match congestion are directly associated with ACL injury risk in professional soccer players.

Limitations

Limitations of this study include the restricted number of analyzable videos due to unrecorded preseason matches, training sessions, and limited streaming coverage before 2023. Only 33 of 65 identified ACL injuries had available video footage for analysis, introducing potential selection bias because publicly available injuries may not fully represent all ACL injuries occurring in MLS athletes. Furthermore, the findings of this study primarily apply to match-play ACL injuries with available video footage and may not generalize to training-related injuries. Injury identification relied partly on publicly available databases and media reports, which may introduce incomplete injury capture or misclassification despite efforts to verify diagnoses through team announcements and news sources. Additionally, prior ACL injury history and laterality could not always be consistently confirmed from publicly available information.

This study also relied on broadcast video analysis, which is inherently subjective and limited by camera angle, frame rate, image resolution, and player occlusion. Although videos were independently reviewed by two reviewers with consensus resolution of disagreements, formal inter-rater reliability analysis was not performed. Estimation of knee valgus, flexion, and loading patterns from broadcast footage lacks the precision of 3D motion capture or biomechanical modeling, which may provide a more accurate assessment of knee angles and stress patterns but requires high-quality footage and multiple viewing angles [26].

Finally, exposure-based denominators were unavailable; therefore, findings regarding field surface, match timing, fatigue, seasonal clustering, and workload should be interpreted cautiously and considered hypothesis-generating rather than causal. This study was also limited to men’s professional soccer players in the United States and Canada, and previous literature suggests that contact-type ACL injuries are more common in males, whereas non-contact injuries are more prevalent in females, although this may not apply specifically to men’s soccer [27-29]. Despite these limitations, this study provides one of the first MLS-specific systematic video analyses of ACL injury mechanisms and offers clinically relevant descriptive data that may help guide future biomechanical and injury prevention research.

## Conclusions

ACL injuries in MLS most commonly occur through non-contact or indirect-contact mechanisms, frequently during defensive pressing or offensive driving movements with the knee in valgus and flexion. Indirect-contact injuries most commonly involve force applied to the upper body, which may contribute to increased stress across the knee joint during directional-change maneuvers. These findings may help generate hypotheses for MLS-specific ACL injury prevention strategies and future biomechanical research focused on high-risk soccer movements.
